# Application of Positron Emission Tomography in Drug Development

**DOI:** 10.4172/2167-0501.1000e128

**Published:** 2012-08-08

**Authors:** Sibaprasad Bhattacharyya

**Affiliations:** Frederick National Laboratory for Cancer Research, USA

Positron Emission Tomography (PET) is a clinically established noninvasive imaging modality [1]. In PET, targeting ligands, or drug molecules labeled with a positron-emitting isotope, typically known as PET-radiopharmaceuticals, are introduced in to the body at a very low concentration (nanomolar or picomolar range), and are not intended to have any pharmacological effect. As the radioisotope decays, it emits a positron that interacts with an electron. This encounter produces a pair of high-energy (511 keV) gamma photons that move in nearly opposite directions. A circular array of detectors captures these photons and determines their source along a straight line of coincidence. These coincidences are forwarded to the image processing unit to generate PET images via mathematical reconstruction procedures (Figure 1). Commonly used PET radioisotopes (^18^F, ^11^C, ^13^N, ^15^O etc.) in clinical and developmental research are very short-lived and cyclotron-produced.

The drug development process is very complex, time-consuming, and costly. The process for bringing a new molecular entity from preclinical discovery to market may take up to 15 years with a cost that may exceed $800 million [2]. Considering the current high failure rate of drugs that enter clinical trials (~ 50% in Phase III), there is a clear need for more efficient and sensitive strategies in the search for useful medicine.

One of such strategy is to find methods for testing more molecules in early, exploratory studies in humans. Drug research now benefits from the rapid development of PET imaging technology, which non-invasively traces radiolabeled molecules directly in the human body. The most straightforward approach is to radiolabel a new potential drug and then to trace its anatomical distribution and binding in the target of interest. As the PET technique is sensitive enough to determine the concentration of radiolabeled drug as low as subpicomolar range, less than a microgram of the radiolabeled drug is sufficient to perform a PET study in humans. This characteristic has significant advantages in the initial phases of drug development. Because the low mass of the radiolabeled drug does not induce drug effects, approval from U.S. Food and Drug Administration may be obtained more quickly, without the extensive documentation required for those drugs that may induce drug effects. This advantage enables researchers to examine a large number of new molecules in humans. Small molecules have faster blood clearance and can reach the target tissue less than an hour or so. Positron emitters ^8^F and ^11^C are ideal radioisotopes because they can be attached with very little or no change in chemical structure or properties of the small drug molecules (Figure 2) [3]. Large molecules (mAb, affibody, large peptides etc.) need a longer time to reach the target tissue and are usually labeled with positron emitting isotopes having longer half-life (^64^Cu, ^89^Zr, ^86^Y etc.). In these cases, a Bifunctional Chelate (BFC) is usually used to attach these radiometals to the drug molecules [1].

In early-phase drug development, a biodistribution study is very important for confirming that the drug molecule reaches the target tissue and does not accumulate in non-target sites, resulting in potential toxicity. A dynamic PET scan measures the concentration-time course of the radiolabeled molecule in the tissue of interest. By measuring the concentration of the radiolabeled drug in the blood, it is possible to use bio mathematical kinetic models to derive estimates of the rate of drug clearance from plasma to tissue as well as the ratio of the concentration of the labeled drug and drug metabolites in tissue to blood. HPLC analysis (radio and UV) of the blood samples of different organs (*ex vivo*) provides additional information on any metabolism of the radiolabeled drug.

In our laboratory, a HER1 receptor-targeting therapeutic antibody (mAb) was recently evaluated [4,5] preclinically by labeling it with ^89^Zr, a positron-emitting long-lived radioisotope. Labeled mAb in nanomolar scale (50-60 μCi) was injected into non-tumor-bearing mice. A biodistribution study revealed that this mAb has minimal (1-5% after 93 h post inj.) uptake in normal, healthy organs. Clearance from blood was very slow, which is normal for fully intact mAb. An *in vitro* study with HER1+ cell line (MDA-MB-468) showed that binding of ^89^Zr-labeled mAb is target-specific. PET imaging with HER1+ (MDA-MB-468) mice xenograft model showed that ^89^Zr-mAb accumulates in tumor. Concentration of the ^89^Zr-mAb in tumor gradually increases with time. Overall, this small preclinical study, showed that this mAb is target specific having very little or no nonspecific binding and can be used for treatment of HER1+ carcinomas safely. Its ^89^Zr-labeled analog can be used as a diagnostic imaging agent to follow or monitor the treatment in a noninvasive way.

Assessing the interaction of a drug molecule with its target in tissue is also a significant aspect of drug development and represents another approach to understanding drug-target pharmacokinetics. This type of study requires a radioligand that binds selectively to the target of interest with very high affinity. This radioligand may be a totally different molecule than the therapeutic drug molecule (to be evaluated) for the same target. The binding potential (BP) of the radioligand is proportional to the specific binding divided by the free concentration of the radioligand [6]. If the PET study is performed after the administration of an unlabeled drug that binds to the same target, the measured radioligand BP will vary with the local free drug concentration. The binding affinity of this drug can be estimated by measuring radioligand’s BP over a range of doses of the unlabeled drug. Determining the relationship between plasma concentration and target interaction for drug molecules can be important if dose limiting toxicities are associated with the drugs. *In vivo* target interaction studies in human by PET can reduce the substantial uncertainty in early-phase drug development. In some cases, target affinity of drug molecules in human is quite different from that measured in preclinical models; in many cases, there is a lack of generally accepted animal models.

Currently, NCI is conducting a clinical trial [7] to understand the safety and effectiveness of the drug edoxifen in individuals with estrogen hormone receptor positive (ER+) solid tumors (breast or others) that have not responded to standard treatment. In this trial, patients will take endoxifen tablets for the cycle of treatment and will be monitored by PET imaging using a 16 alpha-(^18^F-fluoro)-17 beta estradiol [^18^F-FES] radioligand [8], which has high affinity to the same target (ER+). An ^18^F-FES PET/CT scan will be performed at baseline (before treatment) and 1-3 hrs after endoxifen treatment to measure the change in [^18^F-FES] uptake in ER+ tumors. The binding affinity and drug-target pharmacokinetics of endoxifen will be assessed (by PET) from the BP of the radioligand at different endoxifen doses.

Some radiotracers or radioligands can be used for pharmacodynamic studies. Radioligands can measure the concentration of the receptor sites which enables the assessment of the distribution of specific targets that may correspond to their expression. Differences in blood flow between the tissues can be assessed by measuring the concentration/availability of radioligands. Use of ^18^F-fluorodeoxyglucose (FDG) as the PET radiotracer provides an index of enhanced glucose transport and phophorylation in many tumors. Qualitative assessment of FDG-PET signal is used routinely in the clinic as a diagnostic marker for tumors. Quantitative measurements of FDG uptake before and after treatment can define pharmacodynamic effects expressed as a change in glucose transport [9]. Several wellcharacterized PET radiotracers have been used for pharmacodynamic studies (Table 1) [9,10].

Although the major application of PET is primarily in the development of CNS- and oncology-based therapeutics, there is potential for its use in the development of the drugs for other diseases. This technology, along with the array of diverse radioligands, can be used to address several key drug discovery challenges, including biodistribution, absorption, target affinity, plasma binding, metabolism, and dosing. In a clinical context, PET is used to evaluate, stage, and monitor disease quantitatively and objectively. The expansion of this technology will undoubtedly enhance future the drug discovery and development efforts.

This project has been funded in whole or in part with federal funds from the Frederick National Laboratory for Cancer Research, National Institutes of Health, under Contract No. HHSN261200800001E. The content of this publication does not necessarily reflect the views or policies of the Department of Health and Human Services, nor does mention of trade names, commercial products, or organizations imply endorsement by the U. S. government.

## Figures and Tables

**Figure 1:**
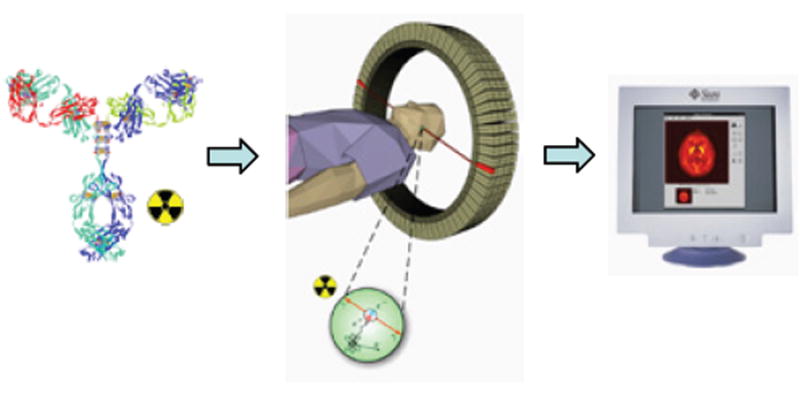
Schematic diagram of PET.

**Figure 2:**
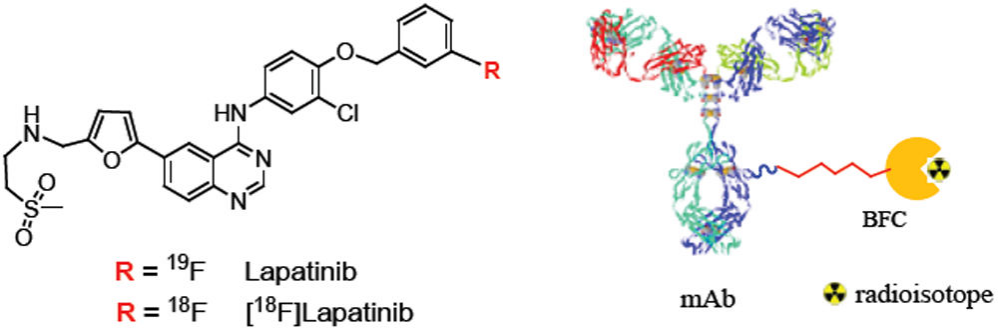
Radioisotope labeled small drug molecule [3] and schematic representation of radiolabeled large molecule (mAb).

**Table 1:** Selected PET radiotracers for pharmacodynamic studies [10].

PET radiotracer	Clinical application
[18F]-fluorodeoxyglucose (FDG)	Glucose uptake and phosphorylation
[18F]-fluorothymidine (FLT)	Tumor cell proliferation
[18F]-DOPA	Presynaptic dopaminergic function
[18F]-sodium fluoride	Bone scintigraphy
[11C]-acetate	Oxidative metabolism
[11C]-methionine	Protein synthesis
[82Rb]-rubidium	Myocardial perfusion
[64Cu]-ATSM	Hypoxia
[13N]-ammonia	Myocardial perfusion
